# Development and internal validation of a risk prediction model for oral frailty in hospitalized older adults with chronic diseases

**DOI:** 10.3389/fpubh.2026.1717485

**Published:** 2026-01-19

**Authors:** Huan Liu, Xinyu Hu, Qingwei Liu, Ming Shao, Xiaohua Huang, Junzhuo Gu, Jingjing Luo, Shichang Fang, Xiubin Tao, Jiating Lin, Ming Zhang, Junkai Dou

**Affiliations:** 1 Department of Hemodialysis, The First Affiliated Yijishan Hospital of Wannan Medical College (Yijishan Hospital of Wannan Medical College), Wuhu, Anhui, China; 2 Department of Cardiovascular, The Second Affiliated Hospital of Wangnan Medical College, Wuhu, Anhui, China; 3 Department of Nursing, Shandong Provincial Hospital Affiliated to Shandong First Medical University (Shandong Provincial Hospital), Jinan, Shandong, China; 4 School of Graduate, Wannan Medical College, Wuhu, Anhui, China; 5 Department of Stomatology, The First Affiliated Yijishan Hospital of Wannan Medical College (Yijishan Hospital of Wannan Medical College), Wuhu, Anhui, China; 6 Department of Nursing, The First Affiliated Yijishan Hospital of Wannan Medical College (Yijishan Hospital of Wannan Medical College), Wuhu, Anhui, China; 7 School of Innovation and Entrepreneurship, Wanna Medical College, Wuhu, Anhui, China; 8 Key Laboratory of Philosophy and Social Science of Anhui Province on Adolescent Mental Health and Crisis Intelligence Intervention, Hefei Normal University, Hefei, China; 9 Nursing Department, Lu’an Hospital of Anhui Medical University, Lu’an, Anhui, China

**Keywords:** factors, nomogram, older adults, oral frailty, risk prediction model

## Abstract

**Background:**

This study aimed to investigate the prevalence and influencing factors of oral frailty, a significant geriatric syndrome, among hospitalized older adults with chronic diseases, and to develop a corresponding risk prediction model to facilitate early screening and intervention.

**Methods:**

From September 2024 to May 2025, we recruited 443 older adult patients with chronic diseases from two tertiary grade A hospitals in Wuhu City, Anhui Province, using a convenience sampling method. Data were collected using a general information questionnaire, the Oral Frailty Index-8 (OFI-8), the FRAIL scale, the Appetite Scale, and the Clinical Physiological Resilience Scale. Binary logistic regression analysis was conducted to identify factors associated with oral frailty.

**Results:**

The prevalence of oral frailty among hospitalized older adult patients with chronic diseases was 69.3%(307/443). Appetite, employment status, age, Clinical physiological resilience, and frailty were the best predictors of oral frailty in hospitalized older adult patients with chronic diseases. These influencing factors were utilized to construct a nomogram model, which demonstrated excellent consistency and accuracy. The area under the curve (AUC) in the training set was 0.805 (95% confidence interval [CI] = 0.753–0.857). In the validation set, the AUC was 0.900 (95% CI = 0.843–0.957). The Hosmer–Lemeshow test values were *p =* 0.465 and *p =* 0.161 (both > 0.05). The calibration curve indicated a significant agreement between the nomogram model and the actual observations. Both the ROC curve and decision curve analysis (DCA) demonstrated that the nomogram possesses outstanding risk predictive performance.

**Conclusion:**

The prevalence of oral frailty is notably high among hospitalized older adult patients with chronic diseases, with key influencing factors including age, employment status, appetite, and clinical physiological resilience. The developed risk prediction model demonstrates good calibration and discriminative ability, supporting its potential use in early screening and targeted intervention of oral frailty in this population.

## Introduction

China is undergoing rapid population aging, accompanied by a rising burden of chronic non-communicable diseases (NCDs) that now pose the foremost threat to the health of older adults (1). In this context, oral frailty—a decline in oral function linked to aging, reduced health literacy, and diminished physical and psychological reserves—has emerged as a critical yet frequently overlooked public health challenge (2, 3).

Poor oral health can lead to increased physical frailty, disability, and a higher mortality rate. A meta-analysis summarising evidence based on 18 studies with a total of 12,932 older adults published by Li et al., the results show that the global prevalence of oral frailty among the older adults population was 24%, while the prevalence in China was 45.9%. Oral frailty has become a serious public health issue threatening the health of the older adults (4). Oral health issues can lead to cognitive decline, reduced social functioning, decreased quality of life, and even increased mortality risk in the older adults (5–7). A cohort study demonstrated that the number of teeth, dental plaque, and marginal bone are associated with all-cause mortality (8). Oral frailty is a dynamic clinical condition (9), where early detection and proactive intervention play an irreplaceable role in slowing or even reversing its progression. The development of risk predictive models for oral frailty to rapidly identify high-risk older adult individuals and reduce their risk of adverse health outcomes holds significant public health importance.

Hospitalized older adults with chronic diseases are particularly vulnerable to oral frailty due to the cumulative effects of chronic illness, polypharmacy, and the hospital environment. However, current research and screening efforts have predominantly focused on community-dwelling older adults, leaving a gap in attention toward the hospitalized population (10). Early identification and intervention for oral frailty in this high-risk group are essential to mitigate adverse health outcomes and improve quality of life.

Frailty, a complex geriatric syndrome characterized by a decline in physiological reserves, results in increased vulnerability and susceptibility of the organism to stressors (11). Frailty is characterized by increased susceptibility, reduced physiological reserve, and decreased resistance to stress. Studies have found that the prevalence of frailty among community-dwelling older adults is approximately 10.7% (12, 13). Recent research indicates a growing global interest in issues related to frailty and oral frailty (14). A systematic review reported that individuals with oral health issues are more likely to experience physical frailty compared to those with good oral health (15).

Appetite is the body’s desire to fulfill its needs, generally divided into three parts: hunger, satiation, and satiety (16). Poor appetite, frequently referred to as the anorexia of aging, can lead to decreased food and nutrient consumption, alterations in dietary preferences, and subsequent weight loss (17, 18). Reduced appetite due to decreased appetite can lead to malnutrition and deficiencies in trace elements. Insufficient intake of calcium, antioxidants, and fatty acids can trigger pro-inflammatory mechanisms, induce periodontitis, and exacerbate oral frailty (19). In recent years, an increasing number of studies have begun to focus on the association between malnutrition and oral frailty and their impact on health outcomes.

Physical resilience is characterized as a dynamic process that signifies an individual’s capability to recover or sustain functionality when confronted with age-related injuries or illnesses (20). Physiological resilience is of significant importance for the older adults in maintaining health levels and coping with health risks (21). The decrease in physical resilience correlates with a deterioration in physical functions and may serve as an indicator of pre-senescence (22). An increased level of physical resilience can lead to favorable clinical results, such as swift recovery of function following significant health challenges like surgical interventions (i.e., regaining physical capabilities). Conversely, inadequate physical resilience might elevate the risk of becoming vulnerable to stressors, leading to negative functional and clinical outcomes, including extended periods of illness or hospitalization, limited physical activity, an elevated likelihood of frailty, and, in severe cases, mortality (23).

The risk prediction model is a valuable tool for assessing the risk of oral frailty in older adult hospitalized patients with chronic comorbidities, as it can effectively integrate multiple factors, enabling early identification of individuals with oral frailty and the formulation of targeted interventions. Therefore, this study aims to identify risk factors associated with oral frailty in older adult hospitalized patients with chronic comorbidities and to develop a nomogram-based prediction model using these risk factors to assess the risk of oral frailty, providing a reference for the formulation of early intervention and management strategies.

## Materials

This multicenter cross-sectional study was conducted from September 2024 to May 2025 at two tertiary hospitals in Wuhu City, Anhui Province: the First Affiliated Hospital of Wannan Medical College and Wuhu Second People’s Hospital. The study followed the Strengthening the Reporting of Observational Studies in Epidemiology (STROBE) guidelines (24).

## Participants

Convenience sampling was employed to recruit eligible older adult inpatients from the departments of endocrinology, ophthalmology, nephrology, and cardiology. This sampling method was chosen due to its feasibility in the clinical setting, facilitating initial data collection on this understudied population within a defined timeframe. However, its implications for the generalizability of findings to broader or different healthcare contexts are acknowledged in the discussion.

Daily screening of electronic inpatient lists was performed to identify potential participants. Their attending physician or primary nurse conducted a preliminary assessment of communication ability and physical condition. Eligible patients were approached by the research team, who explained the study’s purpose, procedures, risks, and benefits. Informed consent was obtained prior to participation.

Inclusion and Exclusion Criteria.

Inclusion criteria were: (1) age ≥60 years; (2) clear consciousness and normal communication ability; (3) permanent residency in Anhui Province; (4) provision of informed consent (written or verbal). Exclusion criteria were: (1) severe visual or hearing impairment; (2) a prior diagnosis of major psychiatric or neurological disorders (e.g., dementia); (3) suffering from a terminal or critically unstable condition.

Sample Size.

The sample size was estimated using Kendall’s method (5–10 times the number of questionnaire items). Accounting for a potential 10% non-response rate, a minimum sample of 400 participants was targeted (25).

## Measurements

### Demographic information questionnaire

Based on the research objectives of the project team, a general demographic questionnaire was developed following a literature review of databases such as PubMed and group discussions. The questionnaire includes: age, gender, place of residence, educational level, previous occupation, marital status, living arrangements, and other information.

### Oral frailty

In this cross-sectional study, the oral frailty of participants was measured using the OF checklist proposed by Tanaka et al. (26). The OFI-8 scale consists of 8 items, encompassing 5 dimensions: dentures, swallowing ability, chewing ability, oral health-related behaviors, and social participation (27). According to the scoring criteria, if the participant answered “yes” to questions 1, 2, and 3, two points were given for the item. If the participant answered “yes” to questions 4 and 5, one point was given for each item. If the participant answered “no” to questions 6, 7, and 8, one point was given for each item. The OFI-8 total score range is 0–11, with higher scores indicating more severe oral frailty in participants. In this cross-sectional study, an OF score of 4 or higher was deemed to indicate the presence of OF in participants.

### Clinical physical resilience

The Clinical Physical Resilience Assessment Scale (CHEES) was employed to measure the participants’ capacity to recover from health-related adversities. This scale was developed in 2024 by Li Jiatong, a scholar from the Department of Geriatrics, Xuanwu Hospital, Capital Medical University, and the Capital Medical University National Clinical Research Center for Geriatrics Disorders (28). The CHEES consists of three dimensions: intrinsic ability, adaptability to change, and external support, comprising a total of 14 items. Each item is rated on a 5-point Likert scale: strongly disagree as 1 point, disagree as 2 points, agree as 3 points, strongly agree as 4 points, and extremely agree as 5 points, with a total score range from 14 to 70. A higher score indicates better physical recovery ability. Liu et al. conducted a reliability and validity test on the CHEES scale among inpatients in the Department of Geriatrics at Xuanwu Hospital, Capital Medical University (29). The research results indicated that the CHEES scale has good validity and reliability.

### Frailty

In this study, the FRAIL scale was used to assess the frailty status of older adult patients with chronic diseases, which was recommended by the Geriatrics Branch of the Chinese Medical Association. The FRAIL scale consists of five components: Fatigue, Resistance, Ambulation, Illness, and Loss of weight (30). The score for each question ranges from 0 to 1, and the total score of the RAIL scale is obtained by summing the scores of the 5 items, with a range of 0–5 points. When the total score of the FRAIL scale is 3 or above, it indicates the presence of frailty (31).

### Appetite

The Simplified Nutritional Appetite Questionnaire (SNAQ) is a widely used brief scale for appetite assessment, particularly valuable in older adult populations. Developed by Wilson et al. in 2005 under the auspices of the Committee on Nutrition Strategies for Community-Dwelling Adults in long-term care facilities in the United States, SNAQ aims to rapidly assess the appetite status of older adults and predict potential malnutrition risks through a concise questionnaire format (32). The SNAQ consists of four items: appetite, feeling of hunger, taste of food, and number of meals per day. Each item is rated on a 5-point scale; the total score range is 4–20 points, and a total score≤14 points is considered indicative of loss of appetite (33). The Cronbach’s *α* for the SNAQ scale in this study was 0.818. The SNAQ, as a simplified version of an appetite assessment tool, not only demonstrates high reliability and validity but has also been widely used and validated among the older adult population in China (34).

### Data collection

Data were collected via structured questionnaires and assessments. For literate and mobile patients, self-administration was encouraged, with investigators available for clarification. For patients with limited literacy or mobility, investigators read the questions aloud and recorded responses based on the participants’ answers, ensuring all responses reflected the participants’ own views.

### Quality control

The following measures were implemented to ensure data quality:

(1) Validated scales with established reliability and validity were used (e.g., Oral Frailty Index-8).
(2) A pilot study with 20–30 patients was conducted to test feasibility and refine procedures.
(3) All investigators received standardized training on instrument use, data collection protocols, and participant communication.
(4) Questionnaires were checked on-site for completeness and accuracy.
(5) Appropriate statistical methods for cross-sectional data were applied during analysis.

### Statistical analysis

In this study, the data entry was conducted using the Wenjuanxing software, and data management and analysis were performed using the SPSS 26.0 statistical software. In this research, all continuous variables were expressed as mean and standard deviation; count variables were expressed as percentages (%). The chi-square test was used to analyze the prevalence of oral frailty among older adult individuals with different characteristics. In the above univariate analysis, independent variables with significance (*p* < 0.05) were retained and entered into a binary logistic regression model using the forward likelihood ratio method to identify the influencing factors of oral frailty in older adult patients with chronic diseases, with an odds ratio (OR) value of >1 is considered a risk factor, and an odds ratio (OR) value of < 1 is considered a protective factor.

To evaluate the efficacy of the risk predictive model, this study employed metrics of discrimination, calibration, and clinical utility. The model’s discriminatory ability was assessed by the area under the receiver operating characteristic (ROC) curve (AUC). Internally, the model’s performance was validated using the bootstrapping method with 1,000 resamples to calculate an optimism-corrected AUC, providing a robust estimate of its generalizability. The calibration curve, along with the Hosmer–Lemeshow test, was utilized to evaluate the agreement between predicted probabilities and actual outcomes. Additionally, decision curve analysis (DCA) was employed to quantify the clinical net benefit. This study utilized R software (version 4.2.2) and the RMS package for statistical analysis. All tests were two-tailed, and a *p*-value < 0.05 was considered statistically significant.

## Results

### Description of the sociodemographic characteristics of the participants

The research involved 443 older adult patients suffering from chronic illnesses who were admitted to the hospital. Table 1 outlines the fundamental demographic details of the subjects. Of these participants, 216 were men (48.8%) and 227 were women (51.2%). The ages of the hospitalized older adult individuals ranged from 60 to 96 years, with a mean age of 71.35 ± 8.07 years.

**Table 1:** Univariate analysis of the participants’ demographics (*N* = 443).

Characteristics	All participants (*n* = 443)	Oral frailty		*χ* ^2^	*p* value
		NO (*n* = 136)	YES (*n* = 307)		
Work status				31.755	<0.001
At work	407 (91.9)	110 (27.0)	297 (73.0)		
Non-work	36 (8.1)	26 (72.2)	10 (27.8)		
Living alone				1.114	0.291
No	427 (96.4)	133 (31.1)	294 (68.9)		
Yes	16 (3.6)	3 (18.8)	13 (81.2)		
Age, years				71.421	<0.001
60–69	198 (44.7)	100 (50.5)	98 (49.5)		
70–79	164 (37.0)	32 (19.5)	132 (80.5)		
≥80	81 (18.3)	4 (4.9)	77 (95.1)		
Gender				0.020	0.887
Male	216 (48.8)	67 (31.0)	149 (69.0)		
Female	227 (51.2)	69 (30.4)	158 (69.6)		
Place of residence				3.505	0.173
Rural	158 (35.7)	40 (25.3)	118 (74.7)		
Town	129 (29.1)	45 (34.9)	84 (65.1)		
City	156 (35.2)	51 (32.7)	105 (67.3)		
Have relatives who are medical staff				0.182	0.670
No	373 (84.2)	113 (30.3)	260 (69.7)		
Yes	70 (15.8)	23 (32.9)	47 (67.1)		
Adhere to regular health check-ups				1.237	0.266
No	303 (68.4)	88 (29.0)	215 (71.0)		
Yes	140 (31.6)	48 (34.3)	92 (65.7)		
Blood transfusion within the last 6 months.				0.014	0.907
No	426 (96.2)	131 (30.8)	295 (69.2)		
Yes	17 (3.8)	5 (29.4)	12 (70.6)		
Monthly per capita household income				13.644	0.009
<1,000 ¥	24 (5.4)	5 (20.8)	19 (79.2)		
1,001–2000 ¥	103 (23.3)	25 (24.3)	78 (75.7)		
2001–3,000 ¥	167 (37.7)	45 (26.9)	122 (73.1)		
3,001–4,000 ¥	118 (26.6)	45 (38.1)	73 (61.9)		
≥4,000 ¥	31 (7.0)	16 (51.6)	15 (48.4)		
Educational level				14.951	0.005
Never attended school	110 (24.8)	27 (24.5)	83 (75.5)		
Primary school	128 (28.9)	29 (22.7)	99 (77.3)		
Junior high school	90 (20.6)	31 (34.4)	59 (65.6)		
High school	80 (18.1)	32 (40.0)	48 (60.0)		
Associate degree or higher	35 (7.9)	17 (48.4)	18 (51.4)		
Number of hospitalizations in the past year				3.544	0.06
1	354 (79.9)	116 (32.8)	238 (67.2)		
≥2	89 (20.1)	20 (22.5)	69 (77.5)		
Frailty				53.865	<0.001
No	315 (71.1)	129 (41.0)	186 (59.0)		
Yes	128 (28.9)	7 (5.5)	121 (94.5)		
Appetite loss				53.204	<0.001
No	174 (39.3%)	88 (50.6%)	86 (49.4%)		
Yes	269 (60.7%)	48 (17.8%)	221 (82.2%)		

### Univariate analysis of risk factors for Oral frailty in older adults

The data analysis of this study ultimately included 443 hospitalized older adult patients with chronic diseases, among whom 136 (30.7%) did not exhibit symptoms of oral frailty, while 307 (69.3%) did. There were significant differences between the oral frailty group and the non-oral frailty group in terms of age, employment status, frailty status, appetite, educational level, and per capita monthly household income (*p* < 0.05). See Table 1.

### The correlation between frailty, appetite, clinical physiological resilience, and oral frailty

**Figure 1:**
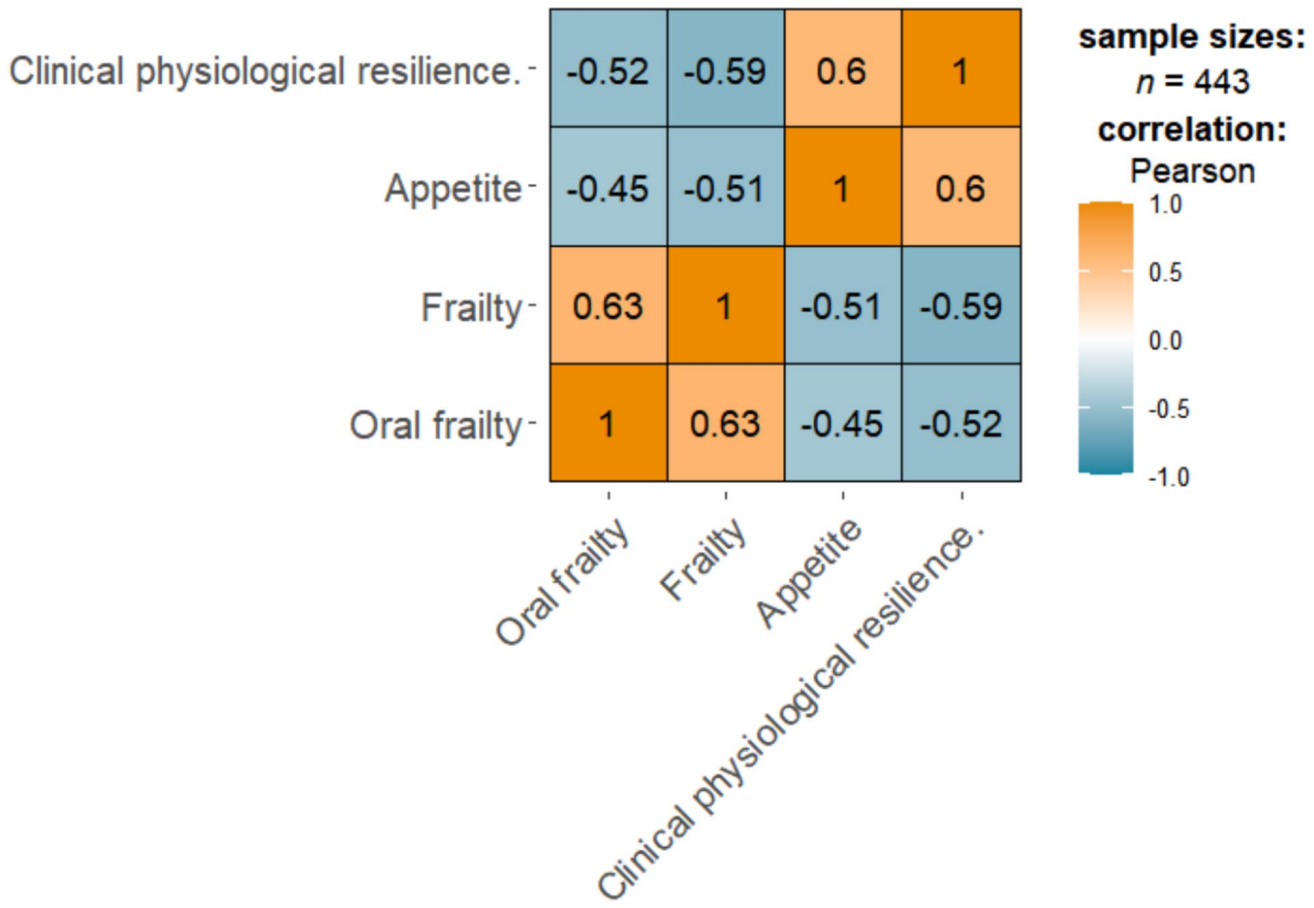
The correlation between frailty, appetite, clinical physiological resilience, and oral frailty.

Figure 1 shows the correlation between oral frailty scores and frailty, appetite, and clinical physiological resilience. There was a significant positive correlation between frailty (*r* = 0.63) and oral frailty (*p* < 0.01). In addition, there was a significant negative correlation between appetite (*r* = −0.45), clinical physiological resilience (*r* = −0.52), and the oral frailty score (*p* < 0.01).

### Factors associated with oral frailty in binary logistic regression analysis

Table 2 and Figure 1 present the factors associated with oral frailty in older adult patients with chronic diseases. Binary logistic regression analysis identified greater frailty severity (OR = 3.621, 95% CI 1.521–8.621), decreased appetite (OR = 1.809, 95% CI 1.055–3.102), and older age (70–79 years: OR = 2.522; ≥80 years: OR = 7.040; reference: 60–69 years) as risk factors for oral frailty. Currently being employed (OR = 0.293, 95% CI 0.121–0.708) and higher clinical physiological resilience (OR = 0.935, 95% CI 0.895–0.977) were protective factors.

**Table 2:** Binary logistic regression analysis of factors associated with oral frailty.

Indices	*β*	S.E.	Wald	*P* value	OR	95% CI	Tolerance	VIF
Appetite loss	0.593	0.275	4.649	0.031	1.809	1.055–3.102	0.589	1.672
Work status	−1.228	0.450	7.434	0.006	0.293	0.121–0.708	0.939	1.065
Age group			19.990				0.755	1.325
70–79	0.925	0.268	11.885	0.001	2.522	1.491–4.268		
≥80	1.952	0.560	12.142	< 0.001	7.040	2.349–21.103		
Clinical physiological resilience	−0.067	0.022	9.059	0.003	0.935	0.895–0.977	0.536	1.865
Frailty	1.287	0.443	8.449	0.004	3.621	1.521–8.621	0.556	1.065
Constant	2.788	1.096	6.471	0.011	16.244			

### Development of risk predictive models

**Figure 2:**
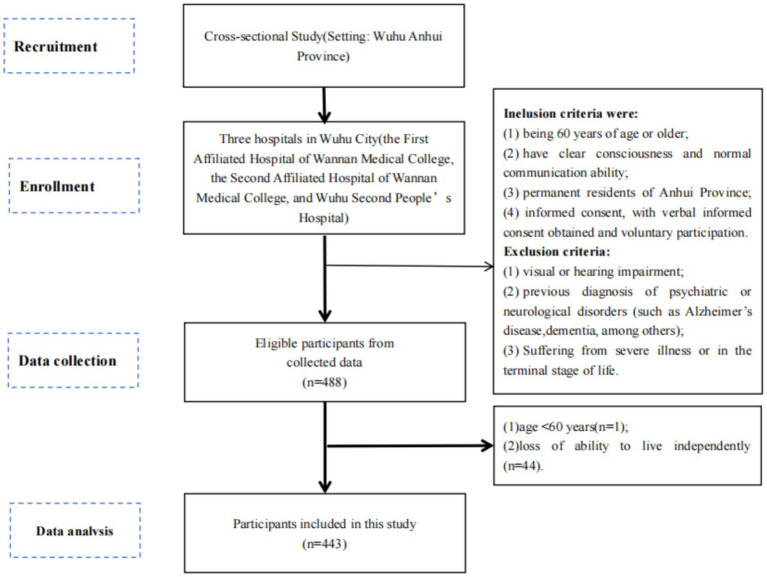
Flowchart of participant selection.

**Figure 3:**
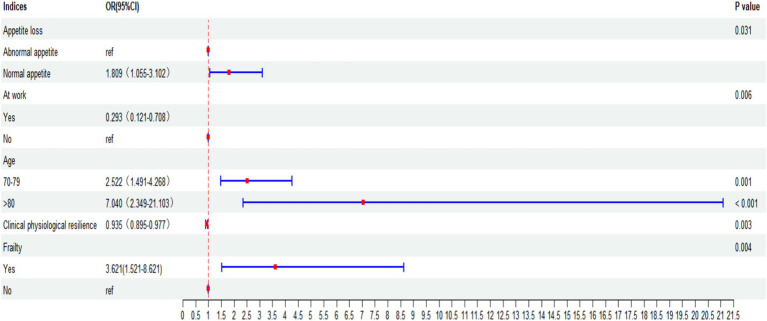
Forest plot of factors influencing oral frailty.

Based on the five influencing factors (Age, Frailty, Appetite loss, State of work, and Clinical physiological resilience) screened through univariate and multivariate analysis, the research team utilized the “RMS” package in R software to construct a nomogram risk prediction model for the occurrence of chronic disease hospitalization or oral frailty in the older adults (Figure 2). In this nomogram model, it is necessary to determine the position of each variable on its respective axis and draw a line on the points axis to obtain the score corresponding to that risk factor. The total score is calculated by summing the scores of each factor. For example, for a patient with decreased appetite (61 points), not working (46 points), presence of frailty (86 points), aged 70–79 (71 points), and clinical physiological resilience of 48 points (36 points), the total score is 300 points, corresponding to a predicted probability of approximately 0.9 (Figure 3).

### Validation of the risk prediction model

**Figure 4:**
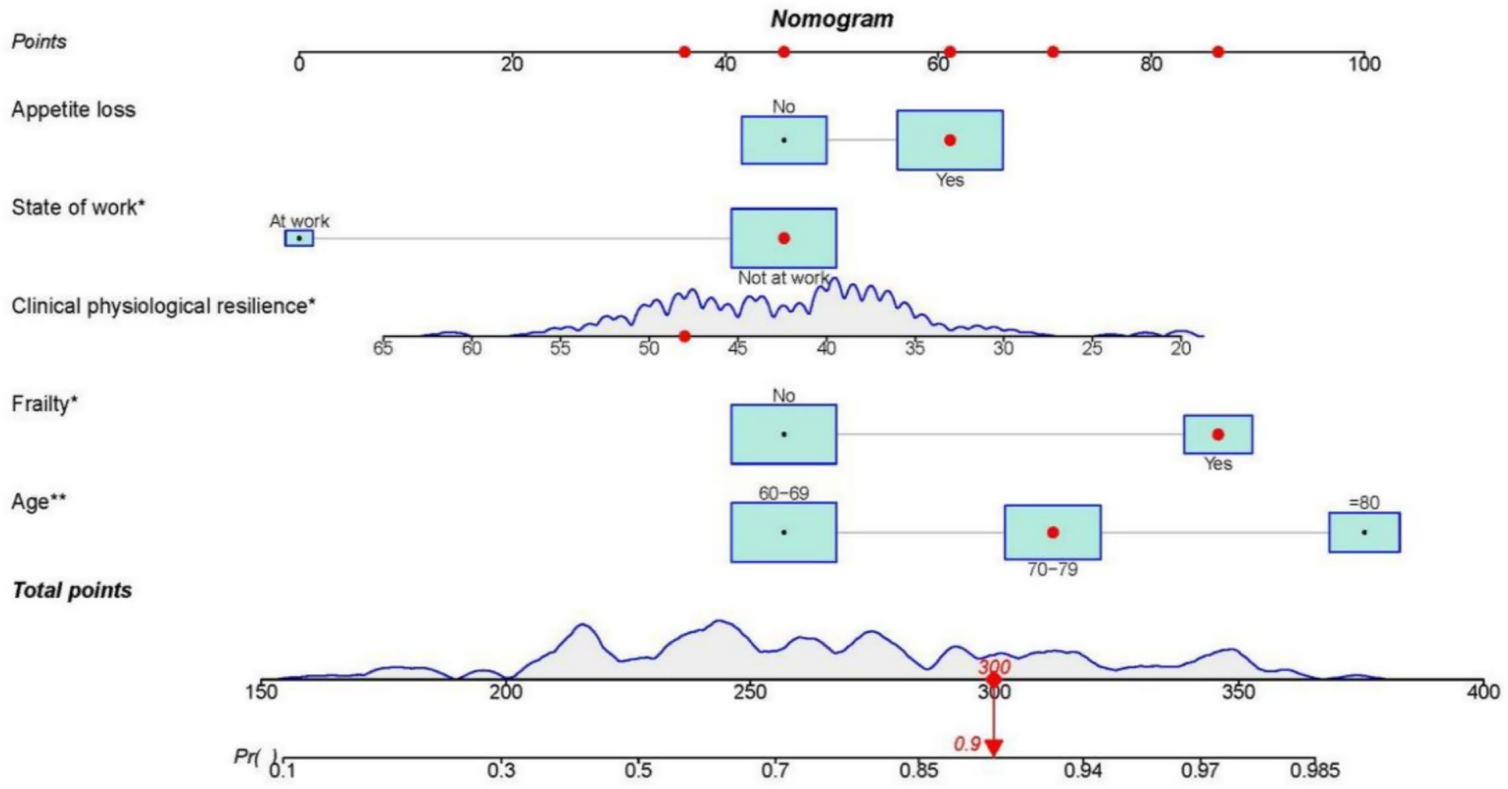
Nomogram for predicting the risk of oral frailty in hospitalized older adult patients with chronic diseases.

The model demonstrated good discriminative ability in both the training set (AUC = 0.805, 95% CI: 0.753–0.857) and the validation set (AUC = 0.900, 95% CI: 0.843–0.957), indicating its effectiveness in distinguishing between individuals with and without oral frailty (Figure 4).

### Calibration of the risk prediction model

**Figure 5:**
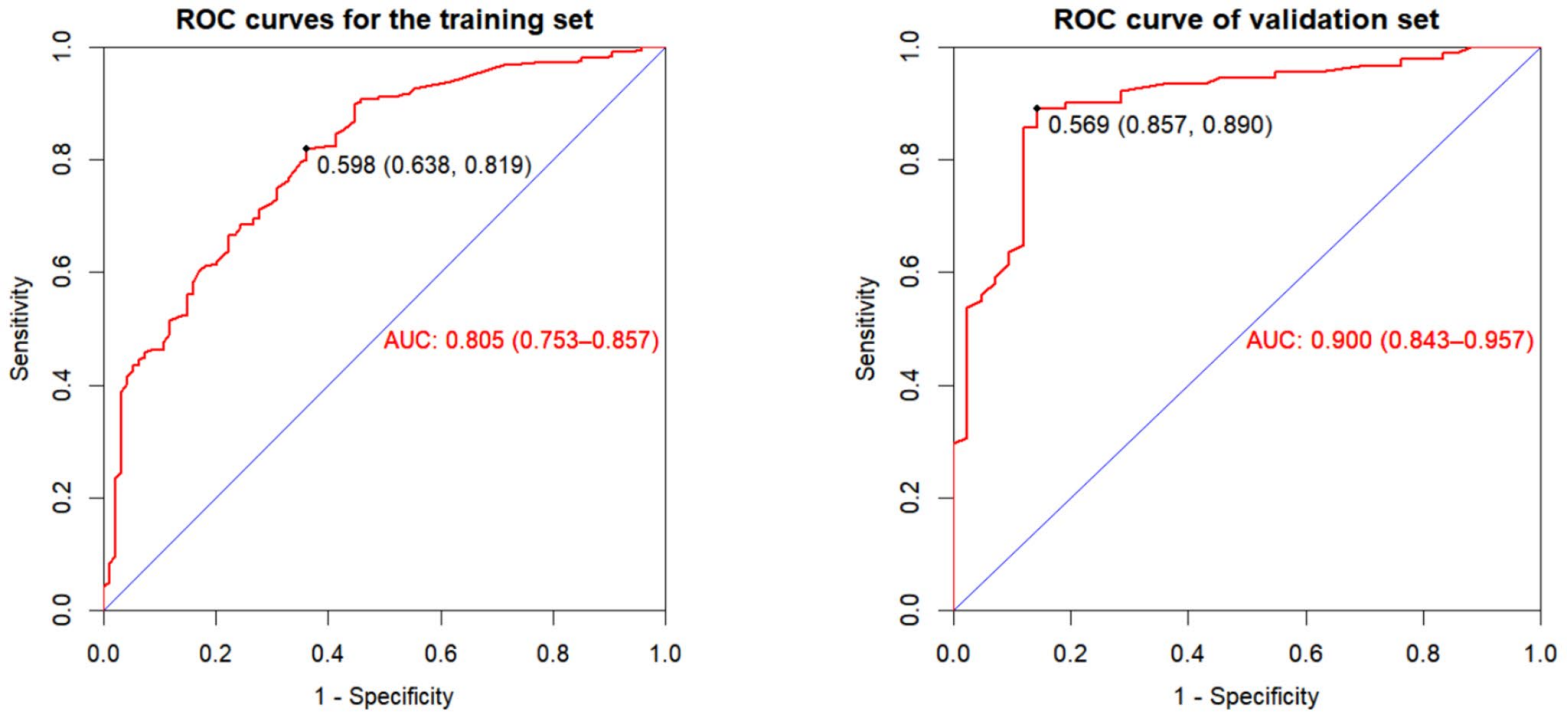
ROC curve of the oral frailty risk model in training and validating cohorts.

**Figure 6:**
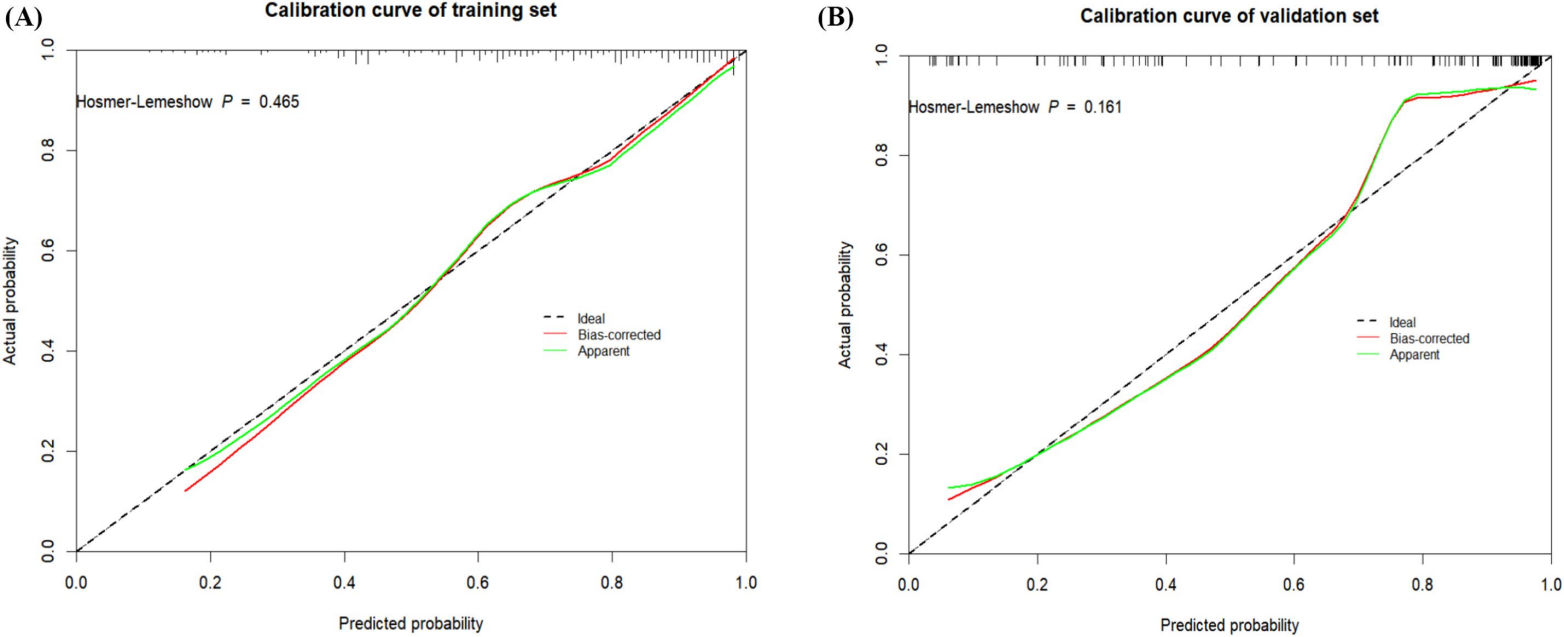
**(A)** Calibration plot for the train dataset. **(B)** Calibration plot for the validation dataset.

Calibration was assessed using the Hosmer–Lemeshow test and calibration plots. The model showed good fit in both the training (*χ*^2^ = 7.679, *p* = 0.465) and validation sets (*χ*^2^ = 11.785, *p* = 0.161), with Brier scores of 0.158 and 0.123, respectively. These results confirm excellent agreement between predicted probabilities and observed outcomes (Figures 5, 6).

### Evaluation of clinical validity

**Figure 7:**
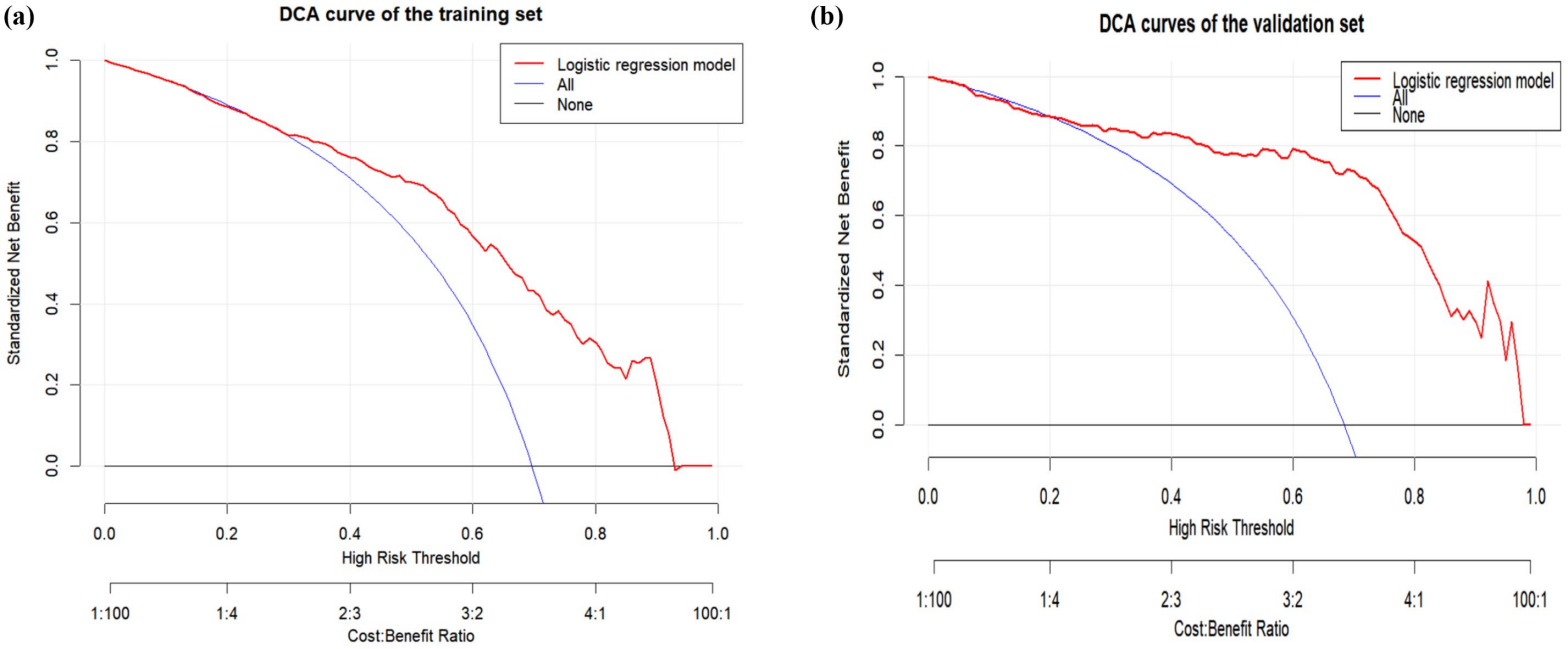
The DCA of the risk predictive model for oral frailty in older adult hospitalized patients with chronic diseases: **(A)** Training set and **(B)** validation set.

To evaluate the clinical validity of the nomogram, we conducted a DCA. The DCA of the training and validation sets demonstrates that the nomogram exhibits a higher net benefit level than “no intervention” and “full intervention” when the probability of oral frailty occurrence is within the broader ranges of 0.30 ~ 0.90 and 0.25 ~ 0.99 (Figures 7A,B). It shows superior overall net benefits across a wide range of practical threshold probabilities, highlighting the model’s excellent clinical applicability and practicality.

### Development of web-based nomograms

**Figure 8:**
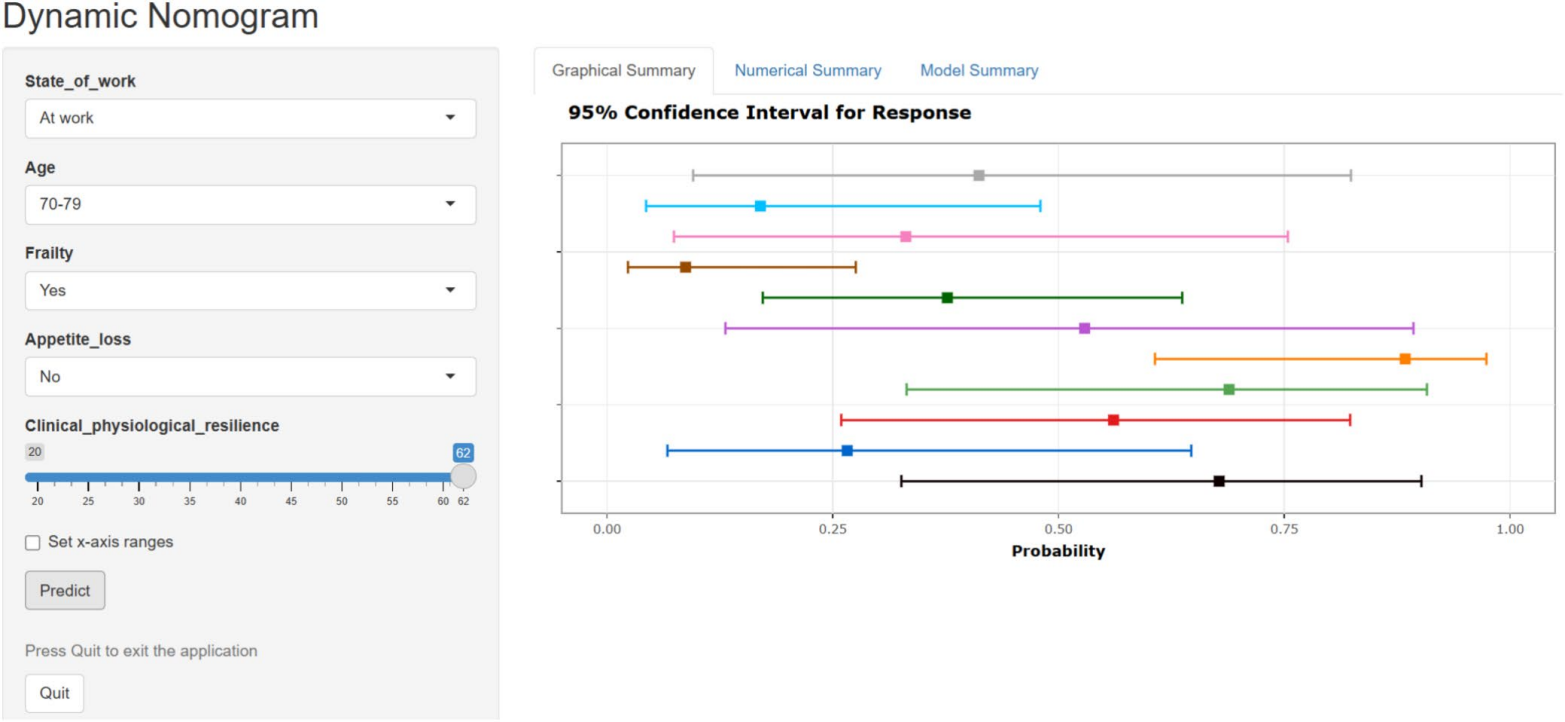
Web-based nomogram for oral frailty in older adult chronic disease inpatients.

To promote the widespread application of the nomogram model in clinical practice, the research team utilized the R software and the “DynNom” package from the Web development platform to successfully develop a web-based nomogram for predicting the risk of oral frailty in older adult hospitalized patients with chronic diseases.^1^ By transforming the nomogram into an online risk calculator, personalized prediction values for the risk of oral frailty in older adult hospitalized patients with chronic diseases were generated, significantly enhancing its application convenience in clinical settings (Figure 8).

## Discussion

This study identified a high prevalence (69.3%) of oral frailty among hospitalized older adults with chronic diseases in China, markedly higher than rates reported in community-dwelling older adult populations (35, 36). This disparity underscores oral frailty as a severe, yet under-addressed, complication within the inpatient setting, likely exacerbated by the cumulative burden of chronic conditions, polypharmacy, and associated systemic inflammation (37).

Five factors were independently associated with oral frailty: frailty, loss of appetite, older age, unemployment, and lower clinical physiological resilience. The strong link between physical frailty and oral frailty suggests a shared pathway of multisystem decline, where reduced mobility may impair oral hygiene and muscle function involved in chewing and swallowing (38, 39). Appetite loss creates a vicious cycle; it contributes to malnutrition that weakens oral tissues, while poor oral health further discourages adequate food intake (40–44). Age-related physiological decline naturally increases vulnerability (45, 46). Employment likely acts as a proxy for socio-economic advantage, better healthcare access, and sustained social engagement, all protective for oral health (47–49). Most notably, clinical physiological resilience emerged as a novel protective factor. This suggests an individual’s intrinsic capacity to recover from stressors may help buffer against the functional decline characteristic of oral frailty (50, 51).

### Clinical and public health significance

The high prevalence (69.3%) of oral frailty identified in this study underscores its significance as a common and pressing health issue among hospitalized older adults with chronic diseases. From a public health perspective, these findings highlight the urgent need to integrate oral frailty assessment into routine geriatric care within hospital systems.

### Public health contribution and implementation

Screening and Prevention: The developed nomogram serves as a practical, preliminary risk assessment tool that can facilitate early identification of high-risk inpatients. This supports the potential for instituting routine, low-cost screening protocols in hospital admission assessments, enabling timely referrals and preventive interventions before functional decline progresses.

Multidisciplinary Care Pathways: The model’s predictors (e.g., appetite, frailty, physical resilience) are non-dental indicators routinely accessible to nurses, physicians, and other frontline clinicians. This promotes a multidisciplinary approach, where non-dental healthcare professionals can play a pivotal role in initial screening, fostering collaboration with dental specialists for comprehensive care.

Health System Planning: Recognizing oral frailty as a key marker of vulnerability can inform hospital management policies, such as tailored nutritional support, oral hygiene assistance programs, and staff training protocols. This aligns with broader goals of improving patient outcomes and optimizing resource allocation within geriatric services.

## Limitations

However, this study has several potential limitations. First, this research is derived from a cross-sectional, single-center study using convenience sampling, which may limit the generalizability of the study to some extent. Second, the self-reported nature of the OFI-8 scale may lead to an underestimation of oral frailty among older adult patients with chronic diseases. Finally, this cross-sectional study was only internally validated and was not externally validated. Therefore, our future research efforts will focus on further external validation at multiple centers to fully explore predictors of oral frailty and effective intervention methods.

## Conclusion

From the perspectives of clinical intervention and public health importance, given the high prevalence of oral frailty among older adult patients with chronic diseases, it is strongly recommended to incorporate oral frailty assessment into the routine evaluation items for hospitalized older adult patients. Through targeted oral health education and interventions promoting healthy lifestyles, we can, to some extent, prevent or delay oral frailty and improve patients’ overall health status and quality of life. In this study, we discovered for the first time a negative correlation between clinical physiological resilience and oral frailty among older adult patients with chronic diseases in China. These findings underscore the importance of healthcare professionals conducting regular comprehensive assessments of oral frailty in older adult patients with chronic diseases, to facilitate early screening of those at risk of oral frailty. Additionally, targeted and comprehensive oral frailty care measures should be implemented for different patients to prevent or slow down the progression of oral frailty, ultimately improving their overall oral health and promoting healthy aging.

## Data Availability

The raw data supporting the conclusions of this article will be made available by the authors, without undue reservation.

## Footnotes

1
https://lqwlqw.shinyapps.io/oral_frailty_web/
